# The basic principles of migration health: Population mobility and gaps in disease prevalence

**DOI:** 10.1186/1742-7622-3-3

**Published:** 2006-05-04

**Authors:** Brian D Gushulak, Douglas W MacPherson

**Affiliations:** 1 Migration Health Consultants, Inc., Vienna, Austria/Cheltenham, Ontario, Canada; 2 Faculty of Health Sciences, McMaster University, Hamilton, Ontario, Canada

## Abstract

Currently, migrants and other mobile individuals, such as migrant workers and asylum seekers, are an expanding global population of growing social, demographic and political importance. Disparities often exist between a migrant population's place of origin and its destination, particularly with relation to health determinants. The effects of those disparities can be observed at both individual and population levels. Migration across health and disease disparities influences the epidemiology of certain diseases globally and in nations receiving migrants. While specific disease-based outcomes may vary between migrant group and location, general epidemiological principles may be applied to any situation where numbers of individuals move between differences in disease prevalence. Traditionally, migration health activities have been designed for national application and lack an integrated international perspective. Present and future health challenges related to migration may be more effectively addressed through collaborative global undertakings. This paper reviews the epidemiological relationships resulting from health disparities bridged by migration and describes the growing role of migration and population mobility in global disease epidemiology. The implications for national and international health policy and program planning are presented.

## Introduction

The relationships between disease, travel and migration have historical roots that continue to influence modern medical activities [1]. Traditional medical approaches dealing with migrant health have focused on the recognition, identification and management of specific diseases, illnesses or health concerns in mobile populations at the time and place of their arrival [2]. These activities have often been based on the principles of protecting the recipient population through policies of exclusion directed at the migrant or arriving traveller. Derived from the historical practices of quarantine, similar processes continue in a modern context through immigration medical screening [3] and border control practices intended to reduce threats to public health or to mitigate potential impacts on healthcare services.

The epidemiological analysis of illnesses and disease in migrants is most commonly approached in one of two ways in receiving countries. The first is to consider the health issue of concern in terms of the status at the time of migration, while the second is to study the evolution of the health characteristic over time [4]. The reference population for the first analytical approach is normally the host or receiving population, while the reference group for the second approach can be either the host population or a comparison cohort at the migrants' place of origin (see table 1).

**Table 1:** Epidemiological approaches to health and migration

**Time Period**	**Epidemiological Characteristic**	**Reference Population**	
*Arrival Phase (Point Prevalence)*	Prevalence		Host/Receiving population
*Post Arrival Phase (Point Prevalence or Longitudinal Studies)*	Incidence/Prevalence	Similar non-migrating cohort at migrants' place of origin	Host/Receiving population

The quarantine-associated historical basis of migration health practices has ensured that much of the interest in health and migration has been directed towards communicable diseases [5]. Commonly, migrant medical screening focuses on conditions differentially prevalent between the migrant and host population, such as tuberculosis [6], leprosy [7], or syphilis [8]. Medical screening has been used to quantify and document aspects of health and disease in migrant cohorts, most often in relation to national public health statistics. Over time, these studies have described some of the immediate and long-term impacts of population movement in individual migrant receiving nations.

Recently, the growing international importance of migration has stimulated new interest in other aspects of migrant health. In addition to communicable diseases, attention is now focused on pre-existing non-infectious diseases [9] and other health domains, including behaviour [10], morality [11] and genetic or ethnic profiles [12] in migrant populations. Epidemiological studies now involve chronic illnesses [13] such as malignancies [14], renal failure [15] and severe cardiac disease [16], as well as mental and psychosocial health [17] and maternal and child health [18]. Lifestyle-associated health issues, including tobacco use, alcohol consumption and substance abuse, are also being examined in relation to the process of migration in some migrant receiving countries [19].

As migrant demographics often vary between receiving nations, international comparisons involving the pooled analysis of several host nations would be challenging in interpretation and of questionable utility. However, interest in the global implications of the epidemiological aspects of migration is growing [20]. The increasing desire for improved information is, however, complicated by the greater diversity now manifest in modern migrant populations. In additional to traditional immigrants, current mobile populations are often comprised of several other cohorts that are not similarly distributed between migrant receiving nations. Those other groups include refugees and asylum seekers, temporary migrants such as international students and migrant workers, and complex groups of irregular or illegal migrants, including those who have arrived through smuggling or trafficking.

The current volume and diversity in migration often exceeds the scope and intent of the traditional methods used to assess and manage health issues in immigrants. As a consequence of this more diverse demographic and fluid environment, the perspectives derived solely from traditional immigrant medical screening practices may be limited. These limitations may be overcome by applying population health principles to the study of migration, a process that may result in observations that are more applicable to immigration health policy and programme development [21].

A population health-based approach considers the relationship between migration and health as a progressive, interactive process influenced by temporal and local variables. The observations are less related to the administrative mechanics of migration [22] and more sensitive to the driving forces that cause people to migrate. A population-based approach also facilitates the consideration and study of the long-term consequences of movement between locations with different health determinants and health outcomes. Use of this approach supports the examination of the issues from a global perspective. Such methodologies are already in use in the health sector. The Global Fund approach to tuberculosis, malaria and HIV similarly represents an integrated, inter-regional action plan in the face of persistent global health challenges [23].

As presented below, considering migration-relevant diseases in terms of population-based risk may be more relevant to disease control programs than time-of-entry screening for individual conditions. Evaluating the migration and mobility history of populations has the additional advantage of supporting longitudinal analysis of health characteristics. This context is important in the consideration of illnesses and diseases with long latency or delayed diagnostic time [24], an area beyond the scope of traditional time-of-entry immigration screening. It also facilitates investigation of the acquisition of positive and negative health attributes by migrants following arrival [25], which is also of increasing interest in migration health. Finally, population-based methods reflect the nature and role of globalisation and can be used to support and rationalise strategies to deal with health challenges at their source.

The population health approach to migration health is based on the standardised examination of two factors: (1) sustained disparate health environments and (2) the movement of populations between regions of differential prevalence of health indicators and outcomes.

### The dynamics of health disparities

Some diseases or illnesses are sustained by differences that are purely geographic or environmental in origin. In other situations, differences in health outcomes, and the factors that determine or influence health outcomes, result from more complex interactions. The environment [26], socio-economics, genetics and biology, and behavioural factors influence population measurements of disease prevalence individually and in combination.

Examples of environmentally-limited diseases include vector-borne conditions, for which environmental factors determine the distribution of disease transmission, as observed in the global epidemiology of malaria, Chagas' disease, yellow fever and West Nile Virus. Environmentally-related non-communicable disease epidemiological disparities include micronutrient deficiencies [27] and geographically-defined exposure risks, such as health outcomes related to extreme weather or altitude [28]. Movement of the population out of the risk environment (i.e. African refugees in Europe and North America and malaria) or establishment of disease transmission outside of the usual environmental constraints (i.e. West Nile Virus in North America) will impact on the epidemiology of the condition in the receiving region and on the local population health outcomes.

Social and economic influences can be significant factors in the creation and maintenance of differences in health and disease outcomes between populations. Poverty, education, housing and nutrition are directly related to disease prevalence and illness outcomes [29]. The capacities and capabilities of medical and health sectors can affect health through the availability, accessibility [30] and affordability of health promotion, disease prevention and treatment services [31,32]. Additional factors that influence health risks and outcomes include language skills [33], behavioural and cultural practices [34,35], such as the use of tobacco, dietary practices and population norms for body mass and physical exercise [36].

Migrants and other mobile populations reflect the health characteristics of their place and environment of origin and carry several of these characteristics with them when they move [37,38]. In addition, migrants are also subject to other specific influences that may affect their health. These factors result from the process of migration itself, for example, during the travel phase between origin and destination. This is frequently observed in refugees, displaced persons and disadvantaged migrant populations such as trafficked or smuggled persons [39-41], and includes events such as trauma and torture [42,43]. Other migration-specific health influences are observed in migrant worker populations [44,45], the children of migrants [46] and returning travellers who have been visiting family and friends [47]. A demographic and mobility process approach to these considerations is presented in table 2.

**Table 2:** The impact of different health environments and the phases of population mobility

**Occurrence**	**Examples**	**Consequence at Destination**
*Pre-departure existing medical condition*	- prevalence of endemic disease - level of development - access to care - availability of care	Arriving population displays health indicators of origin: • Differing incidence and prevalence of illness • Differences in awareness of and use of healthcare services: • preventive • promotional • diagnostic • therapeutic
*Health impacts during migration*	- trauma (physical-psychosocial) - deprivation - violence - exposure - injury	Some populations display greater prevalence of illness resulting from torture, trauma, abuse and exposure • Refugees • Refugee claimants or asylum seekers • Trafficked/smuggled migrants
*Health impacts arising after arrival*	administrative/legal limits - poverty - language culture - occupational risks	Awareness of and use of healthcare services in migrant populations may be limited by immigration status, poverty, language and culture Working conditions may be associated with health risks: • Migrant agricultural labor • Commercial sex workers • Illegal workers • Trafficked migrants
*Health consequences of return travel*	Health environment at origin may have changed - health systems improvements or declines Children born to foreign-born parents have no exposure to risks present at origin	Populations making return journeys to place of origin (particularly children born at new destination) may be at increased risk of disease or illness: "Visiting friends and relative" travellers - Locally born children of foreign-born parents

The impact of genetic and biological determinants of health and disease may be intuitively self-evident. However, in non-endemic regions these influences, and their linkages to population mobility, may be poorly appreciated in the early phases of migration due to lack of awareness, knowledge or experience in the healthcare delivery sector [48].

Disparities in health determinants and disease outcomes are not absolute, but change over time. This temporal variability adds an important dimension of complexity to the analysis and investigation of migrant health concerns, which can affect cohort comparability. Economic and social environments can change rapidly in the modern world. If those changes influence health determinants, consequential changes in health outcomes can be observed over relatively short periods of time. For example, in the thirty years following 1965, the difference between life expectancy for males in the United Kingdom and Russia increased by more than ten years (range from 3.6 to 15.1 years) [49]. Basic public health improvements such as the provision of adequate, safe drinking water, improved sewerage and housing can significantly reduce the incidence and prevalence of diseases of major public health importance in the space of less than a generation [50]. Similarly, conflict, environmental change, natural disasters and population growth can result in new risk exposures and acquisition of adverse health outcomes over short time periods [51]. Genetic admixture and behavioural characteristics of individuals and populations that impact on health outcomes can also occur singly and in combination with other determinants vary over time.

These rapid temporal changes influence the epidemiology of health disparities. If they take place against a background of sustained or growing migration, they may also influence the interpretation of longitudinal and comparative studies involving migrants. Health outcomes for current departing migrants may differ significantly from previous cohorts originating at the same location, as health indicators change over time at the migrants' place of origin. Depending on the nature of the local changes, either improved or worsening health characteristics in current migrant cohorts may be observed. Examples of this ''time phase'' phenomenon can be seen in the improved population health indicators of several Asian nations that have occurred following recent trends in large-scale migration from Asia to Europe and North America. Some of these changes are significant, as indicated by the changing mortality data due to hypertensive cardiac disease in Korea. During the interval between 1984 and 1999, the age-adjusted mortality for hypertensive cardiac disease in Korean men decreased by 92% (from 51.6 to 4.1/100,000) and 84% for women (from 34.1 to 5.3/100,000) [52]. By contrast, examples of diminishing levels of good health indicators can be observed in some Central and Eastern European nations in the period following the dissolution of the Soviet Union [53].

### Modern migration and population mobility

While migration has always been a fluid process subject to change, these changes must now be assessed in terms of the rate of change and global magnitude of population movement. During the past 50 years, the process of migration and concomitant movement of other mobile populations has been markedly influenced by:

1. The decolonialisation of many nations, including those in Africa, the Middle East, Asia, Latin America and the Caribbean;

2. Large refugee movements following conflicts and civil disturbances, including South East Asia, the Balkans, Central America and Central Africa; *and*

3. The political, social and economic consequences of the collapse of the former Soviet Union.

As a result of these events, between 1960 and 2000 the legal and administrative restrictions on the ability to travel, work and move internationally have changed for hundreds of millions of individuals. This has been associated with a profound shift in the demography of people on the move and the nature of migration itself [54]. In traditional migrant-receiving regions such as Australia and North America, patterns of migrant origin have shifted from Europe to source countries in Asia, Africa, Central and South America and the Middle East [55] in the timespan of little more than one generation.

This evolution has not been limited to regulated, traditional immigration and emigration. It has also involved refugee and humanitarian movements and an increase in irregular arrivals (refugee claimants, asylum seeking, smuggling and trafficking in humans). Complex humanitarian emergencies are often associated with large population displacements of refugees, humanitarian evacuees and other displaced populations. Unlike refugee movements before the Second World War that were often passive, modern international attention and efforts are frequently directed at assisting the international relocation of vulnerable migrant populations. International organisations such as the United Nations High Commission for Refugees [56] and the International Organization for Migration [57], as well as other infrastructures, now support and facilitate the selection, movement and resettlement of these populations. These activities are now global in scope and planning, involve an increasing number of nations and, when triggered, take place more rapidly than historical refugee resettlements [58].

Against this backdrop of political, social and civil societal change, the nature, speed and access to international travel has also undergone marked evolution. Travel patterns have been affected by changes in transportation technology, accessibility, and affordability. Growth in air travel has functionally reduced previous limits on the rapid international movement of large numbers of individuals. In 1960, there were approximately 70 million international journeys globally. The number of similar international journeys in 2004 was in excess of 760 million [59]. The high volume of international travel supports greater population exchange and return flows between migrant origin and destination locations.

Increased international travel has also been an integral component of the growing process of globalisation. The progressive integration of global economic and communications sectors has been accompanied, if not preceded by, a corresponding growth in the international demand and flow of labour and manpower. The International Labour Organization estimates the foreign-born migrant labour force to be nearly 90 million persons worldwide [60]. In several locations, there is a repetitive flow of workers between regions of origin and regions of employment. Some of these movements are regular and organised. However, modern population pressures and economic push-pull factors related to marked global differences in opportunity are increasingly associated with irregular population flows facilitated by either smuggling or trafficking of those seeking a better life [61].

Another recent series of factors affecting population mobility and migration has resulted from geo-political changes such as the collapse of the former Soviet Union [62]. The resulting economic, social and political consequences have both direct and indirect impacts on the demography and movement patterns of migrants to Western Europe, parts of Middle-East Asia and the Americas [63]. Current immigration, family reunification and return dynamics in Europe and Central Asia are profoundly different from those of only two decades previously. These changes have been manifested in both voluntary migration, such as that observed in migrants from the former Soviet Union to Israel [64], as well as the flow of refugees and those displaced by regional conflict, as exemplified in the Balkans in the mid to late 1990s [65].

### The epidemiological consequences of mobility across differentials in disease prevalence

In much of the developed world, infections that were historically significant causes of illness and death have decreased in incidence and prevalence or been eliminated. This was accomplished through sanitation, immunisation, antibiotic therapy, and improved healthcare and public health practices. By the end of the last millennium, several globally important infections had reached the point where they were no longer of public health significance in economically advantaged areas of the world. In some areas of the developed world, domestic transmission of serious infectious diseases, such as measles [66] and polio [67], has been eliminated. This is far in advance of what was observed or possible in the developing world. This can create enormous differences in the prevalence of certain conditions between locations. In a mobile world, travellers and migrants crossing these prevalence gaps can become the source for outbreaks of these diseases [68].

The creation of such prevalence differences is not limited to communicable diseases. The ability to treat and manage non-infectious diseases in highly developed regions of the world likewise differs from the situation in many developing regions. Access to and use of complex and costly interventions, such as cancer treatment, organ system support, transplantation and extensive pharmacotherapy vary according to levels of national economic development. These disparities in social investments in health services availability and population access to healthcare are associated with several differential inter-regional health outcomes, including premature death and increased morbidity in the developing world [69].

In the absence of extensive international travel, population mobility and migration, the effects of differences in disease prevalence would have limited global significance. Nations and regions would strive to improve their domestic health capacities and reduce the domestic burden of disease and illness within their population much as they have done throughout history. However, expanding travel and migration across these prevalence differentials now function as an increasing, population-based bridge between the disparities. The net result is the global extension of what was predominantly a local risk.

The global extension of regional epidemiological differences can have marked impact on the local epidemiology of disease. Examples include tuberculosis, sexually-acquired infections, Chagas' disease and strongyloidiasis, for which foreign-born migrants from hyper-endemic areas represent the majority of national case burden in low incidence or non-endemic nations [70-73]. Similar patterns of epidemiological evolution exist for other long-term infections such as hepatitis B and C, as well as HIV/AIDS in some European nations, where these diseases are reported to be more common in foreign-born migrants than in native-born residents [74-76].

Migration-related epidemiological influences on domestic disease patterns for migrant-receiving nations are also observed for non-infectious diseases [77]. Migrants arriving from less developed regions of the world may have had less access to preventive care, health promotion programmes, and diagnostic or therapeutic interventions for illness and disease. Cancer detection programs [78] and periodic health examinations may not be commonly accessible in many populations. Access to healthcare providers and basic services are unequally distributed or subject to limited availability in many places. Similar differences in availability can be observed for smoking and substance abuse prevention programmes, programmes to detect and manage vitamin or micronutrient deficiencies, promotion of dental health and programmes to manage genetic or biological conditions [79]. As a consequence, migrants may present with disease in more advanced stages than normally observed by providers in the destination country [80].

In considering migration in a population health context, it is important to note that not all of the migration-associated epidemiological changes relate to situations where the migrants are less advantaged than the host population. In terms of lifestyle-related non-infectious illnesses, many migrants and new arrivals display health parameters that are better than those of the receiving population [81]. Over time and resulting from a variety of factors including acculturation, diet and behavioural changes, immigrant populations may acquire and display common adverse health indicators more similar to those of the receiving population [82]. Ironically, some of the beneficial factors arriving with immigrants may be adapted locally to the general benefit of the host population, but may be lost over time in immigrant population and their locally-born children.

### Temporal effects of migration on local health and disease epidemiology

Migration-associated influences on the epidemiology of disease have both immediate and long-term effects on host country health indicators due to differentials in disease prevalence, as well as magnitude factors associated with population census shifts (because of the number of migrants and births to the foreign-born cohort). For diseases of rare or limited occurrence, particularly where national incidence has been reduced to very low levels, the presentation of even a single case can have important implications locally and internationally [83]. This can result in a heightened perception of threat to the public health of the local population and increase concerns regarding capacity and response of healthcare service delivery. Recent examples of this include the global public health control efforts resulting from the 2003 SARS events [84,85], avian-to-human influenza transmission [86,87], periodic outbreaks of viral haemorrhagic fevers [88] and the impact of HIV/AIDS cases in Europe and North America [89,90] acquired abroad.

The continued arrival of new residents from high prevalence areas will contribute to the existing disease base of low incidence migrant-receiving locations. Over time, as observed with regard to globally prevalent but non-uniformly distributed diseases such as tuberculosis [91], imported cases among migrants and other mobile populations can come to represent the majority of the case load in the recipient nation. In these situations, where domestic epidemiology comes to reflect global disease distribution through the process of migration, health policy implications become apparent. Long-term healthcare policy and planning in migrant-receiving nations will have to encompass an international and more global focus to be effective. Reliance on historical, domestic epidemiology for policy implementation in these nations will have limited relevance when disease volumes and case-burdens originate beyond the mandate and jurisdiction of national prevention and control efforts [92]. For example, hepatitis A infection associated with population mobility in some western nations has raised concerns for the need of national domestic immunisation programs to deal with what is, in fact, the international persistence of the disease [93].

In terms of non-infectious diseases, migration-associated pressures result from the need to provide service delivery in culturally or linguistically sensitive programmes for the prevention or treatment of illness in migrant communities. While uncomplicated in principle, the provision of health promotion or prevention advice, such as that recently recommended for asthma and atopy in migrant populations [94], can be both a logistical and resource challenge [95].

The reintroduction of diseases into low incidence locations through migration coupled with the growth of new populations with wide linguistic and cultural diversity can create difficulties in recognition, diagnosis and treatment [96]. In some situations, delayed treatment can have important consequences. These consequences may directly affect the patient [97], influence demands on programs and, for some contagious conditions, challenge the public health management of the exposed population. The growth and ease of travel, in conjunction with the increased numbers of those who are mobile, continues to raise the likelihood of the global extension and presentation of many diseases and illnesses. Maintaining sufficient degrees of clinical suspicion as well as laboratory diagnostic expertise, capacity and access can be costly and complicated. Growing needs for cultural and linguistic competencies in the health sector increases this complexity [98]. Such preparedness requires the education, training and maintenance of competence of healthcare providers and the associated infrastructures for diagnosis and care [99] (see table 3).

**Table 3:** Health service issues resulting from international population mobility

**Immediate Term: Response to Imported/Introduced Illness**
Continued/Enhanced need for Clinical/Laboratory Capacities for imported diseases • Provision of services and facilities • Sustained laboratory capacity for rare or exotic diseases • Continuing education of healthcare providers in global health issues • Post-graduate training in low probability but high impact diseases • Maintenance of competency in international health issues and response • Contingency planning and exercise testing of plans • Global health programs in universities and medical/nursing schools • Development of specialized reference centers and international networks

***Long Term: Response to Growing Foreign Born Population Component***

Increasing Demands for Service/Access Provision of appropriate diagnostic and treatment services • Modification of training programs for health providers • Translation and interpretation services • Increased migration of health professionals from migrant source regions • Training/certification of migrants who have linguistic skills • Cultural awareness and sensitivity programs and training

### Future impact of population mobility on global health

Negative health outcomes resulting from migration and population mobility can be expected to increasingly exert major influences on both national and global health planning. Mobility is a basic and fundamental component of the rapidly expanding globalisation process. Analysis suggests that the volume of immigration, travel and the migration of labour are expected to remain at current levels or increase for the foreseeable future. At the same time, current regional and global health disparities are anticipated to remain or increase, despite the international desire and efforts to reduce them. Global efforts aimed at reducing global disparities and impacts of disease and ill health, such as attempts to achieve the Millennium Development Goals [100], are currently underway. These are long-term initiatives that will take time, resources and extensive effort to achieve and maintain. The longer inter-regional disparities in health and health outcomes persist, the longer they will continue to influence the health of migrants, as well as mobile and non-mobile populations, and the greater the challenge and cost will be to effect control of these conditions.

Historically, the majority of the health issues associated with migration, or occurring as a result of migration, have been managed at the national level. This has been accomplished through either immigration health activities or exclusion, or as a component of other domestic health programs. Demand for these services will remain in some locations due to specific situational issues or particular migratory movements. Nationally mandated immigration health screening [101] and the management of health issues associated with the Hajj [102] are two examples of those situations. In other areas, the evolution of travel and migration has reduced the effectiveness of many national, point-of-arrival activities. New patterns of population mobility require reconsideration of the practicality and viability of border-health inspection for exclusion or containment strategies. Some nations with large immigration medical programs maintain specific screening or intervention programs for targeted diseases such as tuberculosis, syphilis and HIV/AIDS. Accumulating evidence suggests that more effective outcomes could be obtained through interventions focused on disease control efforts in source nations rather than reliance on arrival screening alone [103]. The globalisation of risks – manifest by the mobility of large numbers of individuals flowing across and between disparities in health environments and disease prevalence – will require increased investment in globally-focused resources and management commitments, as opposed to inward-looking national management strategies and programs.

The increased recognition of the importance of these issues has already resulted in reconsideration of international policy and program activity [104]. Global response initiatives have been designed, and in some cases implemented, primarily as a result of concerns regarding the potential adverse population health outcomes posed by certain infectious disease threats. International collaboration is underway to track and monitor disease in the global context. Some of these activities are represented by the internationalisation of epidemiological surveillance, reporting and response reflected in the recent revisions to the International Health Regulations [105].

It is evident that health program and policy planning processes need to anticipate and manage the future impacts of population mobility [106]. In several western nations, migrants and other mobile populations represent sizeable and growing population sectors. Over time, the specific health needs and characteristics of these mobile population cohorts will exert greater influence on the health sector in the receiving host nations [107]. The influence of genetic and biological factors in health and disease will increase in parallel with the size of diverse migrant populations. At the same time, the impact and influence of the health environment and epidemiology of disease at the migrants' place of origin will continue to be reflected in both migrants and the children of migrant parents [108] long after the period of immigration.

Addressing these challenges at the national level will require some changes in the epidemiological context that is applied to prospective policy development and programme management (see table 4). In a globalised world in which travel and migration represent the experience of a large and growing population cohort, they can be expected to assume a standard role as a determination factor influencing many health outcomes [109]. In a manner similar to age, sex, genetics, biology, behaviour, and educational and wealth attainment, the mobility history at both the individual and population levels will need to become a routine consideration in healthcare policy, planning, education, training and service delivery [110].

**Table 4:** Health policy issues resulting from international population mobility

• National point-of-arrival activities – for example, immigration medical screening programs for specific targeted disease at the airport – will become less effective, more costly, and increasingly irrelevant • International intervention programs for specific diseases at the migrants' country of origin may be more effective than national intervention programs dealing with low incidence diseases • Mobility will become a more important determination factor influencing many health outcomes, along with age, sex, genetics, biology, behaviour and educational and wealth attainment • Mobile population health policy frameworks will increasingly require integration and harmonisation at all jurisdictional levels with international economic, trade and security approaches

Similar population-based approaches are already in use in some of the national and international infectious disease surveillance and monitoring systems described above [111]. The analysis and interpretation of the information collected by those systems has demonstrated the importance of migration-related travel in terms of imported tropical infections [112]. The extension of the collection of the travel and mobility history to non-infectious disease surveillance is both logical and supported by preliminary study.

### Summary

At the national and international level, the epidemiological outcomes and issues related to migration can be seen to result from the predictable effects of population flows between and across regional disparities and disease prevalence differentials. The growing number of migrants of diverse nature is bridging existing and developing gaps in health outcome indicators. The dynamics of migration and population mobility are evolving at a rate that creates health challenges for existing policy and programme frameworks that differ from those observed in historic migratory movements [113].

The net result is an ongoing globalisation of health influences and indicators currently relevant at both national and global level. The epidemiological impact of population mobility is now evident in a considerable amount of infectious disease surveillance information [114] and similar impacts can be anticipated for non-infectious illnesses in immigration receiving nations [115].

As long as global health disparities and prevalence differentials exist, national health programs and policies in migrant receiving nations will continue to be challenged by illness and disease arising beyond their jurisdiction. National control and regulatory systems alone will be unable to extend their immediate mandate or authority to the source of the problem. To be effective, the management of health issues resulting from population mobility will require an integration of national and global health initiatives for both infectious [116] and non-infectious [117] disease conditions.
